# Prevalence of Malaria Among Individuals Living With Lymphedema in Kamwenge District, Western Uganda

**DOI:** 10.7759/cureus.93691

**Published:** 2025-10-02

**Authors:** Vicent Mwesigye, Joanita Berytah Tebulwa, Benson Musinguzi, Twinomujuni Muzafaru, Henry Zamarano, Charles Nkubi Bagenda, Edgar Mulogo, Frederick Byarugaba, Itabangi Herbert

**Affiliations:** 1 Department of Medical Laboratory Sciences, Mbarara University of Science and Technology, Mbarara, UGA; 2 Department of Nursing, Mbarara University of Science and Technology, Mbarara, UGA; 3 Department of Medical Laboratory Sciences, Muni University, Arua, UGA; 4 Department of Microbiology and Parasitology, Mbarara University of Science and Technology, Mbarara, UGA; 5 Department of Community Health, Mbarara University of Science and Technology, Mbarara, UGA; 6 Department of Microbiology and Immunology, Busitema University, Mbale, UGA

**Keywords:** chronic swelling, elephantiasis, kamwenge district, lymphedema, malaria, podoconiosis, public health, rural health, uganda

## Abstract

Lymphedema, also known as elephantiasis, is a long-term and often debilitating condition characterized by the progressive swelling of limbs due to poor lymphatic drainage. While lymphatic filariasis, a mosquito-borne disease, is a common infectious cause globally, non-infectious forms such as podoconiosis resulting from prolonged exposure to mineral-rich soils are also prevalent in many low-resource, endemic regions. In areas where both malaria and lymphedema occur, malaria may further affect individuals already suffering from chronic swelling. In Kamwenge District, Western Uganda, lymphedema is a recognized public health concern, yet the contributing factors remain poorly understood. This lack of clarity complicates effective diagnosis, treatment, and disease control. This study aimed to assess the presence of malaria among individuals living with lymphedema in this setting.

A cross-sectional study was conducted among 154 individuals with clinically confirmed lymphedema, recruited through purposive sampling from Rukunyu Hospital and surrounding communities. Data collection involved structured interviews, physical examinations, and venous blood sampling. Malaria infection was determined through microscopic examination of blood smears.

Of the 154 participants, 71.4% were female, with an average age of 54.7 years. *Plasmodium falciparum* was detected in 3.3% (n=5) of the individuals. The majority (96.8%) had bilateral lower limb lymphedema and resided in rural areas, primarily engaged in subsistence farming.

The presence of malaria among individuals with lymphedema highlights the need for integrated healthcare approaches in areas where multiple parasitic diseases are endemic. Although malaria was detected in a small portion of participants, its occurrence alongside lymphedema underscores the importance of continued disease surveillance, targeted interventions, and community education to support affected populations.

## Introduction

Lymphedema, particularly affecting the lower limbs and commonly referred to as “elephantiasis,” is a chronic and debilitating condition resulting from impaired lymphatic drainage, leading to the accumulation of protein-rich interstitial fluid [1,2]. This stagnation of lymph causes progressive swelling, fibrosis, and skin changes such as dermal thickening, papillomatosis, and acanthosis [3]. Although the condition can arise from a variety of causes, it is generally classified as either primary, due to congenital or genetic defects in the lymphatic system, or secondary, resulting from external factors such as infection, surgery, or trauma [4,5].

Globally, lymphatic filariasis (LF), a mosquito-borne parasitic disease, is a leading cause of secondary lymphedema. It is caused by Wuchereria bancrofti, Brugia malayi, and Brugia timori, which can obstruct lymphatic vessels over time [6,7]. Malaria, caused by *Plasmodium spp.*, remains one of the leading causes of morbidity and mortality in Uganda. In 2023, malaria remained a major global health challenge, with an estimated 263 million cases reported, an increase from 252 million in 2022, and approximately 597,000 associated deaths, translating to a mortality rate of 13.7 per 100,000 people at risk [8]. The World Health Organization (WHO) African Region continued to carry the heaviest burden, accounting for 94% of all malaria cases and 95% of malaria-related deaths. In Uganda alone, more than 16 million cases were recorded in 2023, leading to roughly 2,793 deaths, the majority of which occurred in children under the age of five.

Additionally, LF and malaria are endemic in Kamwenge District in Western Uganda [7,9]. The co-infection of these diseases poses unique epidemiological, clinical, diagnostic, and public health challenges that warrant comprehensive investigation. The presence of both malaria and lymphedema in Kamwenge District, Western Uganda, poses a significant public health challenge, as malaria infection in individuals with lymphedema may contribute to more severe disease outcomes and complicate clinical management. A 2018 sero-survey in Busiriba sub-county reported a circulating filarial antigen (CFA) prevalence of approximately 1.0% among 101 participants, confirming the continued presence of LF in the area [7]. Malaria remains highly prevalent nationally, accounting for 30-50% of outpatient visits, 15-20% of hospital admissions, and 9-14% of inpatient deaths, with an estimated incidence of 478 cases per 1,000 population annually. Although local malaria prevalence data for Kamwenge are limited, these national figures suggest a substantial burden. Malaria infection in individuals with lymphedema is particularly concerning, as their compromised immune function may increase susceptibility to more severe forms of the disease. Furthermore, the chronic inflammatory state associated with lymphedema can complicate the clinical presentation of malaria, making diagnosis and timely treatment more difficult [10]. These challenges highlight the importance of integrated surveillance and management approaches in regions where both conditions are prevalent. In regions like Kamwenge District, Uganda, malaria remains a major public health concern and may be underdiagnosed among individuals with lymphedema due to overlapping symptoms and limitations in standard diagnostic methods.

In resource-limited settings, malaria diagnosis through microscopy or rapid diagnostic tests remains the standard approach. Identifying malaria in individuals with lymphedema is particularly important, as coexisting infections may influence disease severity and progression. Understanding the presence of malaria in this population helps inform clinical management and supports efforts to prevent further complications associated with chronic conditions like lymphedema.

Although some research has examined the individual impact of malaria, few studies have investigated its role in individuals already living with lymphedema. In malaria-endemic areas like Kamwenge District, Uganda, infection with *Plasmodium falciparum* may further complicate the clinical picture and influence the progression of lymphedema [11]. Despite this potential interaction, limited research has explored the burden of malaria among individuals affected by lymphedema. Gaining insight into this relationship is essential for informing targeted interventions and improving health outcomes in the region.

This study assessed the occurrence of malaria among individuals living with lymphedema in Kamwenge District, Western Uganda.

## Materials and methods

Study design

This study was a cross-sectional survey.

Study population and setting

Individuals with lymphedema who had resided in Kamwenge District for over three months were selected using purposive sampling.

Inclusion and exclusion criteria

Participants were eligible for inclusion if they showed signs of lymphedema, regardless of age, and were either receiving treatment at Rukunyu Hospital or had been recruited from the broader Kamwenge District community. Informed consent was required from adult participants and emancipated minors, while for children over seven years, both caregiver consent and the child’s assent were necessary. The study excluded individuals who were currently undergoing treatment.

Sample size calculation

The sample size for this study was calculated using the Kish and Leslie formula (1965),

n = Z2 P(1-P)

r2

Where:

Z is the standard normal deviate corresponding to a 95% confidence level (Z = 1.96),

P is the estimated prevalence of lymphedema in Kamwenge district, which was 34.7% (0.347), based on a recent study [7]

r is the desired margin of error, set at 8% (0.08).

By substituting these values into the formula, the calculation was as follows:

n = 1.96² × 0.347 × (1 - 0.347) / 0.08² n = 136 participants

Based on the Kish and Leslie (1965) formula, the initial calculated sample size was 136 participants. To account for potential non-response and incomplete data, a 10% adjustment was applied, increasing the minimum required sample size to 150 individuals with lymphedema. These participants were to be recruited from Rukunyu Hospital. Ultimately, a total of 154 participants were successfully enrolled in the study.

Laboratory procedures

Sample Collection and Processing

A vein was identified and punctured using a sterile vacutainer needle and holder, with blood collected into an ethylenediaminetetraacetic acid (EDTA) vacutainer tube. Following standard venipuncture protocols, about 2 mL of venous blood was drawn from each participant into a clean, labeled collection tube. The median cubital vein was typically used, and the area was disinfected with cotton wool soaked in 70% alcohol, then allowed to air dry. The collected blood samples were transported to the laboratory, where blood smears were examined for malaria parasites.

Malaria Testing

Blood films were prepared, stained, and examined to detect malaria parasites. Both thick and thin smears were made on clean glass slides and allowed to air-dry. The dried smears were then stained with 10% Giemsa solution for 10-15 minutes, followed by rinsing with buffered water at pH 7.2. After drying, the slides were examined under a light microscope using oil immersion at 100x magnification. Thick films were primarily used for parasite detection, while thin films facilitated the identification of *Plasmodium* species. Thick and thin blood smears are widely regarded as the gold standard for diagnosing malaria, as they allow for both detection and differentiation of *Plasmodium* species. The sensitivity of thick smear microscopy typically ranges from 75% to 90%, depending on parasite load and the skill of the microscopist, while its specificity is usually above 90%. Although thin smears are less sensitive, they play a crucial role in accurately identifying the *Plasmodium* species involved.

Data Management and Statistical Analysis

Data were double-entered by the principal investigator into Microsoft Excel (Redmond, USA) and subsequently exported to STATA version 17 for cleaning and statistical analysis. The Shapiro-Wilk test was used to assess the normality of age distribution, which was found to be normally distributed (p = 0.08797). Age was summarized as mean ± standard deviation. Categorical variables were described using frequencies and percentages and analyzed using Fisher’s exact test.

## Results

Socio-demographic characteristics of study participants

Out of the 154 participants enrolled in the study, the majority were female (110, 71.4%), while 44 (28.6%) were male. The average age was 54.7 years (±15.6). Age distribution showed that 1 participant (0.6%) was under 10 years, 6 (3.9%) were between 11 and 24 years, 45 (29.2%) were aged 25 to 49 years, and 102 (66.2%) were over 50 years old. Regarding educational background, 79 participants (51.3%) had not completed primary education, 68 (44.2%) had attained primary-level education, and 8 (7.9%) had reached secondary education. Marital status distribution showed 44 (29.1%) were single, 71 (47.0%) married, 26 (17.2%) separated or divorced, three (2.0%) cohabiting, and 7 (4.6%) widowed. Ethnically, the majority were Bakiga (132 participants, 85.7%), followed by Banyankole (3 participants, 1.9%), and 19 (12.3%) from other ethnic groups. Most participants (114, 75.0%) were peasants or farmers involved in agriculture and livestock rearing, with 137 (91.3%) living in rural areas. Lymphedema predominantly affected the lower limbs, with 149 participants (96.8%) presenting with bilateral lower limb involvement. Additionally, 16 participants (11.3%) had discharging wounds (Table 1).

**Table 1:** Socio-demographic characteristics of study participants. *For some variables (marital status, religion, occupation, residence type, and discharging wounds), the total (n) varies due to missing values.

Variable	Total* N=154	
		Gender		
Male	44 (28.6%)	
Female	110 (71.4%)	
Age (years): Mean ± SD	54.70 ± 15.6	
Age groups		
≤ 10	1 (0.6%)	
11 – 24	6 (3.9%)	
25 – 49	45 (29.2%)	
≥ 50	102 (66.2%)	
Education		
None	79 (51.3%)	
Primary	68 (44.2%)	
Secondary	7 (4.5%)	
Marital status		
Single	44 (29.1%)	
Married	71 (47.0%)	
Separated/divorced	26 (17.2%)	
Cohabiting	3 (2.0%)	
Widowed	7 (4.6%)	
Tribe		
Munyankole	3 (1.9%)	
Mukiga	132 (85.7%)	
Others	19 (12.3%)	
Religion		
Catholic	64 (42.1%)	
Protestant	66 (43.4%)	
Moslem	1 (0.7%)	
Pentecostal	11 (7.2%)	
Others	10 (6.6%)	
Occupation		
Housewife	9 (5.9%)	
Business	8 (5.3%)	
Peasant farmer/Grazing	114 (75.0%)	
Student	2 (1.3%)	
Civil servant	1 (0.7%)	
Others	18 (11.8%)	
Residence type		
Rural	137 (91.3%)	
Semi-urban/Trading centre	13 (8.7%)	
Limbs affected		
Lower limbs	149 (96.8%)	
Upper limbs	2 (1.3%)	
All limbs	3 (1.9%)	
Discharging wounds		
Yes	16 (11.3%)	
No	125 (88.7%)	

Malaria test results

Among the 154 participants, *Plasmodium falciparum* was detected in 3.3% (n=5) of the individuals tested.

## Discussion

This study explored the presence of malaria among individuals diagnosed with lymphedema in Kamwenge District, Western Uganda. Out of 154 participants, Plasmodium falciparum infection was detected in 3.3% of cases, indicating ongoing malaria transmission within this population. Although the prevalence was relatively low, the presence of malaria among individuals already burdened by chronic lymphedema has important clinical implications [12]. Coexisting malaria can potentially exacerbate inflammation, weaken immunity, or complicate the clinical management of lymphedema [13], especially in low-resource settings where access to prompt diagnosis and treatment may be limited [14]. This finding aligns with broader concerns in co-endemic regions, where overlapping diseases can influence one another’s progression and outcomes [15].

The results suggest that even in areas where malaria control efforts such as insecticide-treated nets (ITNs) and public health campaigns are active, residual transmission remains a challenge [16]. For individuals with lymphedema, already vulnerable to infections and secondary complications, malaria adds an additional layer of health risk that should not be overlooked in disease surveillance or care planning [12].

Moreover, given the broader burden of non-infectious causes of lymphedema in the region, such as podoconiosis, it is essential to recognize malaria not as a primary cause but as a complicating factor that may worsen health outcomes for those living with this chronic condition [17].

These findings emphasize the importance of integrated disease management strategies that take into account the complex interactions between malaria and other chronic conditions like lymphedema. Strengthening diagnostic capacity, increasing community awareness, and promoting comprehensive care, particularly in rural, high-risk populations, are key steps in improving long-term health outcomes.

Limitations

This study relied on microscopy to detect malaria infections, which, while specific, may miss low-density parasitemia or asymptomatic cases. Additionally, because the study focused solely on individuals with clinically confirmed lymphedema, the findings may not represent the broader population. The cross-sectional nature of the study also limits the ability to assess causality or determine seasonal variation in malaria transmission. Further research, including longitudinal studies and the use of more sensitive diagnostic tools, would enhance understanding of the relationship between malaria and chronic lymphedema.

## Conclusions

The detection of *Plasmodium falciparum* in individuals living with lymphedema in Kamwenge District highlights the need to consider malaria as an ongoing public health concern for this population. While prevalence was low, the presence of malaria in people already affected by chronic swelling points to the importance of integrated healthcare approaches that address both infectious diseases and chronic conditions. Continued surveillance, public education, improved access to timely malaria diagnosis, and tailored interventions targeting high-risk groups are essential in reducing the compounded burden of disease in this setting.
